# Rates of confirmatory HIV testing, linkage to HIV services, and rapid initiation of antiretroviral treatment among newly diagnosed children living with HIV in Ethiopia: perspectives from caregivers and healthcare workers

**DOI:** 10.1186/s12887-022-03784-3

**Published:** 2022-12-26

**Authors:** Alemayehu Bekele, Susan Hrapcak, Jelaludin Ahmed Mohammed, Jemal Ayalew Yimam, Tsegaye Tilahun, Tenagnework Antefe, Hanna Kumssa, Desta Kassa, Semegnew Mengistu, Kelsey Mirkovic, Eric J. Dziuban, Zena Belay, Christine Ross, Wondimu Teferi

**Affiliations:** 1 Ethiopian Public Health Association, Addis Ababa, Ethiopia; 2 Centers for Disease Control and Prevention, Atlanta, Georgia USA; 3 Centers for Disease Control and Prevention, Addis Ababa, Ethiopia; 4 United States Agency for International Development, Addis Ababa, Ethiopia; 5 Amhara Regional Health Bureau, Amhara, Ethiopia; 6 Addis Ababa City Administration Health Bureau, Addis Ababa, Ethiopia; 7 Ethiopian Public Health Institute, Addis Ababa, Ethiopia

**Keywords:** Children, HIV, Confirmatory testing, Rapid ART initiation, Enrollment, Linkage

## Abstract

**Background:**

Successful linkage to HIV services and initiation of antiretroviral treatment (ART) for children living with HIV (CLHIV) is critical to improve pediatric ART coverage. We aimed to assess confirmatory testing, linkage, and rapid ART initiation among newly diagnosed CLHIV in Ethiopia from the perspectives of caregivers and healthcare workers (HCWs).

**Methods:**

We conducted standardized surveys with HCWs and caregivers of children 2–14 years who were diagnosed with HIV but not yet on ART who had been identified during a cross-sectional study in Ethiopia from May 2017–March 2018. Eight health facilities based on their HIV caseload and testing volume and 21 extension sites were included. Forty-one children, 34 care givers and 40 healthcare workers were included in this study. Three months after study enrollment, caregivers were surveyed about timing and experiences with HIV service enrollment, confirmatory testing, and ART initiation. Data collected from HCWs included perceptions of confirmatory testing in CLHIV before ART initiation. SPSS was used to conduct descriptive statistics.

**Results:**

The majority of the 41 CLHIV were enrolled to HIV services (*n* = 34, 83%) and initiated ART by three months (*n* = 32, 94%). Median time from diagnosis to ART initiation was 12 days (interquartile range 5–18). Five children died before the follow-up interview. Confirmatory HIV testing was conducted in 34 children and found no discordant results; the majority (*n* = 23, 68%) received it within one week of HIV diagnosis. Almost all HCWs (*n* = 39/40, 98%) and caregivers (*n* = 31/34, 91%) felt better/the same about test results after conducting confirmatory testing.

**Conclusion:**

Opportunities remain to strengthen linkage for newly diagnosed CLHIV in Ethiopia through intensifying early follow-up to ensure prompt confirmatory testing and rapid ART initiation. Additional services could help caregivers with decision-making around treatment initiation for their children.

## Background

The most recent global estimates from UNAIDS suggest coverage of antiretroviral treatment (ART) in children living with HIV (CLHIV) < 15 years is only 54%, compared to 74% in adults, and Ethiopia’s estimated pediatric ART coverage (40%) is even lower [1]. Although the main barrier to improving ART coverage among children is pediatric case identification, there is a need to better understand linkage to HIV services and ART initiation among newly diagnosed CLHIV. High rates of linkage to ART initiation are critical to achieving UNAIDS 95–95-95 goals by 2030 (95% of people living with HIV are diagnosed, 95% of those diagnosed are on ART, and 95% of those on ART are virally suppressed) [2]. The World Health Organization (WHO) expanded eligibility for ART in the 2016 guidelines to promote universal eligibility for ART for all people living with HIV (the “test and treat” approach), with same-day ART initiation when possible [3]. Early ART initiation among newly diagnosed CLHIV is associated with lower mortality and improved physical and cognitive development [4]. Though test and treat policies have been in place in Ethiopia since 2017, a study conducted in northwest Ethiopia found that only 42% of newly diagnosed adults had same-day ART initiation [5]. However, there is little published data available on timing of ART initiation in CLHIV, or facilitators and barriers to linkage to rapid ART initiation (within 1 week of HIV diagnosis) in the era of universal eligibility in low-resource settings such as Ethiopia. WHO also requires confirmatory HIV testing before ART initiation [6], but there is little published data on implementation of confirmatory HIV testing or its impact on ART initiation in CLHIV. There is also a need to better understand missed opportunities for earlier identification of CLHIV to understand gaps in diagnostic and linkage services. With better data, HIV programs can adapt health systems, policies, and practices to ensure earlier diagnosis and rapid ART initiation to improve pediatric ART coverage.

This study aimed to describe rates of linkage to ART initiation for CLHIV, facilitators and barriers to successful linkage, missed opportunities for earlier diagnosis, disclosure status among newly diagnosed CLHIV, and experiences with implementation of confirmatory testing in children before initiation of ART in CLHIV from the perspectives of caregivers and healthcare workers (HCWs).

## Methods

We conducted standardized surveys with HCWs and caregivers of children 2–14 years diagnosed with HIV not yet on ART that had been identified during a cross-sectional study in Ethiopia from May 2017–June 2018. The details of this study have been described elsewhere [7]. Briefly, this study offered study enrollment to all children 2–14 years of age accompanied by a caregiver and presenting to five service delivery points at participating facilities (medical inpatient wards, malnutrition treatment points, TB clinics, referral of orphans and vulnerable children (OVC), and index testing) in 29 public health facilities in Amhara and Addis Ababa regions. For the OVC entry point, community-based OVC staff notified facility-based study staff when the caregiver and child would present to the facility for HTS and study enrollment. Informed written consent and assent were obtained at the time of study enrollment. After obtaining consent, data were collected on demographic, clinical information, anthropometric data (weight and height), and HIV status. The demographic information was gathered from the caregivers. Clinical information was gathered partly from the caregivers and partly from patients’ records. Anthropometric data and HIV status were collected from patients’ records. All initial and confirmatory HIV testing was done by HCWs following the national testing algorithm as per standard of care at the time of the study [8]. Initial HIV testing was conducted at the presenting entry point at the facility, and all confirmatory testing was conducted at ART clinics prior to initiating ART. CLHIV not on ART at study enrollment were contacted by study staff three months after enrollment were linked to care and treatment services following national HIV guidelines.

Approximately three months after study enrollment, all caregivers of CLHIV not on ART at study enrollment were contacted by study staff by their method of choice (telephone, pre-arranged personal visit to residence, or follow-up appointment at the study facility) and willingness to participate in the follow up study was also asked to take part in the follow up survey. The average duration used to collect the data was 15 minutes. Standardized survey questions collected data on status of linkage to HIV care and treatment services and ART initiation; experience with the process of HIV service enrollment, confirmatory testing, and ART initiation; disclosure status of the child; and potential missed opportunities for earlier diagnosis. When possible, data were verified from available registers and records in the health facilities.

Standardized surveys were also conducted with all eligible HCWs who performed confirmatory HIV testing for children at eight study facilities in Addis Ababa (Yekatit Hospital, Zewditu Hospital, Addis Ketema Health Center and Entoto Fana Health Center) and in Amhara region (Felege Hiwot Hospital, Debre Birhan Hospital, Kobo Health Center, and Shewa Robit Health Center) and who also agreed to participate in the study. These eight facilities were purposively selected based on client load and high number of HIV tests, and additional details on site selection has previously been described [7]. Data collected from HCWs included perceptions and experiences of clinical providers in relation to confirmatory testing of CLHIV before ART initiation.

All data were collected using Android-based tablets. Data collection forms were programed using Open Data Kit (ODK). SPSS version 20 was used to compute descriptive statistics. Descriptive statistics were used to calculate rates and timing of confirmatory HIV testing and ART initiation. Frequencies of enablers and barriers to confirmatory testing, enrollment to HIV services, and ART initiation were determined. Rapid ART initiation was defined as initiation within one week following initial HIV diagnosis. Severe acute malnutrition was defined by a very low weight for height (below -3z scores of the median WHO growth standards) and moderate acute malnutrition was described as wasting or weight-for-height < − 2 z-score and > − 3 z-score for children. Poor health was defined as a child having poor health condition or illness that caused him/her absent from school, frequent visit to health facilities, not feeling well etc. as reported by the caregivers. Missing a lot of school was defined as a child missing many school days or absenteeism because of illness as reported by the caregivers.

### Ethical considerations

This study complied with local and international regulations in regard to Good Clinical Practice, Human Subjects Protection, and institutional review board (IRB) guidelines. Prior to initiation, the protocol was reviewed and approved by the IRB of the Ethiopian Public Health Institute (EPHI), Amhara Regional Health Bureau, Addis Ababa City administration Regional Health Bureau, and the U.S. Centers for Disease Control and Prevention (#6823). Consent and assent forms were translated into Amharic and submitted for IRB approval. All study staff and relevant hospital staff were trained in confidentiality and Basic Human Subjects Protection. Permission was obtained from the respective study facilities to obtain data. In addition, information about the purpose and procedure of the study was explained to all study participants and individuals who provided written consent and assent were finally interviewed. Compensation was made for transport fee that the respondents spent.

## Results

### Participant characteristics

All 41 caregivers of CLHIV that were eligible for the follow-up survey participated in the survey three months after study enrollment (40 newly diagnosed children plus 1 known CLHIV not on ART) (Table 1). Median age of the 41 CLHIV was 9 years (interquartile range (IQR) 6–13). Twenty-one (51%) children were male, and the biological parents of most children were alive (the remaining *n* = 6, 15% reported deceased mothers and *n* = 7, 17% reported deceased fathers). Thirty-six (88%) of the participants were from urban settings and almost half (*n* = 19, 46%) were young adolescents (age 10–14 years). Over half (*n* = 21, 51%) had moderate or severe acute malnutrition. Forty (98%) had no previous history of hospital admission; the one child with a prior hospitalization was the known CLHIV not yet on ART. Fifteen (37%) children were reported to have history of poor health, and recurrent skin problems were found in 23 (56%) of children. Twelve (29%) children reported missing a lot of school.

**Table 1:** Characteristics of children living with HIV not yet on ART in health facilities in Amhara and Addis Ababa regions, Ethiopia, May 2017 – June 2018

Characteristic	Number (***N*** = 41)	Percent (col %)
Timing of diagnosis		
New diagnosis	40	97.6
Previous diagnosis, not yet on ART	1	2.4
Region		
Amhara	25	61.0
Addis Ababa	16	39.0
Entry point		
Index testing	28	68.3
TB clinic	3	7.3
Inpatient	2	4.9
Malnutrition	5	12.2
Orphans and vulnerable children program	3	7.3
Sex		
Female	20	48.8
Male	21	51.2
Age category		
1–4 years	9	22.0
5–9 years	13	31.7
10–14 years	19	46.3
Primary caregiver		
Mother	22	53.7
Father	12	29.3
Sibling	1	2.4
Grandparent	2	4.9
Uncle/aunt	4	9.8
Father’s status		
Alive	33	80.5
Deceased	7	17.1
Unknown	1	2.4
Mother’s status		
Alive	35	85.4
Deceased	6	14.6
Unknown	0	0.0
Nutritional status		
Severe acute malnutrition	18	43.9
Moderate acute malnutrition	3	7.3
Normal nutritional status	20	48.8
Place of residence		
Urban	36	87.8
Rural	5	12.2
Prior hospitalization		
Yes	1	2.4
No	40	97.6
Poor health for the past 3 months		
Yes	15	36.6
No	26	63.4
Recurrent skin problems		
Yes	23	56.1
No	18	43.9
Missed a lot of school due to poor health		
Yes	12	29.3
No	16	39.0
Not applicable	13	31.7

### Confirmatory testing

Confirmatory testing before ART initiation was done in the majority of the children by 3 months (*n* = 34, 83%). Same-day confirmatory HIV testing was conducted in approximately one-half (*n* = 16, 47%) and an additional one-fifth were done within 1 week (*n* = 7, 21%). The median time to confirmatory testing was 1.5 days (IQR 0–14). There were no discordant results between the initial test and confirmatory tests in this study.

The majority of caregivers (*n* = 30, 88%) of the 34 children who received confirmatory testing were satisfied with the experience (Table 3). Most caregivers felt better or the same about their child’s HIV test results when confirmatory testing was done (*n* = 19, 56% and *n* = 12, 35% respectively). Among the caregivers that did not receive confirmatory testing, reasons included: not understanding why the child needed confirmatory testing (*n* = 1), confusion with the initial test results (*n* = 2), HCWs did not offer a confirmatory test (*n* = 1), death of child before confirmatory testing (*n* = 1), ART was not started (*n* = 1), and did not know if the child received confirmatory testing (*n* = 1). Forty HCWs participated in the survey, and the majority stated that they felt better about the testing results and about starting children on ART after conducting the confirmatory testing (*n* = 39, 98% and *n* = 36, 90% respectively) (Table 3).

### Linkage to ART

Among the 41 children, 34 (83%) enrolled in HIV care and treatment services at the time of follow-up (Table 2). Most children (*n* = 28, 82%) enrolled in the same facility that initial HIV testing had been conducted, and the remaining 6 children (18%) were enrolled in other facilities. Of the 34 children enrolled in HIV services, the majority (*n* = 32, 94%) were started on ART; one child died after enrollment in HIV services but before ART initiation, and the caregiver of another child did not see the need for confirmatory HIV testing. The known HIV-positive child not on ART at the time of study enrollment was enrolled in HIV services and initiated ART at the time of follow-up. Date of ART initiation was available for the majority of children that initiated ART (*n* = 31, 97%). Median time from initial HIV diagnosis to ART initiation was 12 days (IQR 5–18 days). Seven (23%) children-initiated ART between 1 and 7 days after diagnosis and only 3 (10%) had same-day ART initiation.

**Table 2:** Linkage of children living with HIV to care and treatment services in health facilities in Addis Ababa and Amhara regions, Ethiopia at three-month follow-up, May 2017 to June 2018

Characteristic	Number (*N* = 41)	Percent (col %)
The child enrolled for HIV care and treatment services		
Yes	34	82.9
No	7	17.1
Location of HIV care and treatment services*		
The same facility that testing was done	28	82.4
Other than the facility that testing was done	6	17.6
Satisfied with your experience at this location*		
Yes	29	90.6
No	3	9.4
Satisfied with the amount of information received about the diagnosis and medical needs of the child*		
Yes	31	96.9
No	1	3.1
The child started antiretroviral therapy (ART)*		
Yes	32	94.1
No	2	5.9
Satisfied with the experience of your child being on ART*so far		
Yes	30	93.8
No	2	6.3
Satisfied with the amount of information received about diagnosis and medical needs of the child*		
Yes	31	96.9
No	1	3.1

Skip patterns excluded caregiver responses

**Table 3:** Caregiver and healthcare worker views on confirmatory testing in children living with HIV in selected health facilities in Addis Ababa and Amhara regions, Ethiopia at three-month follow-up, May 2017 to June 2018

Caregiver perception	Number (***N*** = 34)^**+**^	Percent (col %)
If confirmatory test done before initiation of treatment, the confirmatory test make you feel better about the results		
Yes	19	55.9
No	3	8.8
I felt the same	12	35.3
If confirmatory test done before initiation of treatment, you were satisfied with your experience in the process of retesting of your child to confirm his/her HIV status before initiation of treatment		
Yes	30	88.2
No	4	11.8
I felt the same	0	0.0
Confirmatory HIV test result		
Positive	34	100.0
Negative	0	0.0
**Healthcare worker perception**	**Number (*N*** **= 40)**	**Percent (%)**
Confirmatory testing is important before initiation of ART		
Yes	37	92.5%
No	2	5%
Not sure	1	2.5%
Comfortable discussing confirmatory testing with caregivers		
Yes	39	97.5%
No	1	2.5%
Perceived reaction of most patients about being offered confirmatory testing to confirm HIV status in their children?		
Comfortable	31	77.5%
Did not like it	9	22.5%
Felt better about results after conducting confirmatory testing		
Yes	39	97.5%
No	1	2.5%
Felt better about starting children on ART after conducting confirmatory testing		
Yes	36	90.0%
No	4	10.0%
Confirmatory testing made the caregivers feel better about the results		
Yes	32	80.0%
No	8	20.0%
Task of conducting confirmatory testing was burdensome		
Yes	13	32.5%
No	27	67.5%
Adequate space at ART clinic to conduct confirmatory testing		
Yes	26	65.0%
No	14	35.0%
If no adequate space, where is the best place to conduct confirmatory testing to confirm HIV status of children? (*n* = 14)		
Laboratory	2	14.3%
Voluntary counseling and testing clinic	2	14.3%
Other*	10	71.4%
If space at ART clinic is adequate, who should do confirmatory testing at the ART clinic? (*n* = 26)		
ART provider	18	69.2%
Separate person assigned	4	15.4%
Other	4	15.4%
Current register is adequate to capture the new information		
Yes	22	55%
Not adequate	16	40%
Not available	2	5%

^+^Caregivers of the 7 children that did not receive confirmatory testing did not provide responses to these questions

*Suggestions included separate room at ART clinic, adherence room at ART clinic

**Table 4:** Missed opportunities for HIV testing among 40 newly diagnosed children living with HIV in selected facilities in Addis Ababa and Amhara regions, Ethiopia at three-month follow-up, May 2017 to June 2018

Missed opportunities for HIV testing	***N*** = 40	Percent (col %)
Timing of maternal diagnosis (*N* = 40)		
Prior to/during pregnancy	4	10.0%
During breastfeeding	3	7.5%
After breastfeeding ended	26	65.0%
Don’t know	7	17.5%
If diagnosed after breastfeeding ended, were you offered HIV testing during pregnancy (*N* = 26)		
Yes	3	11.5%
No	18	69.2%
I don’t know	5	19.2%
If offered HIV testing during pregnancy, did you accept testing (*N* = 3)		
Yes	1	33.3%
No	2	66.7%
Infant tested within 2 months of life (for mothers diagnosed prior to/during pregnancy or during breastfeeding, *N* = 7)		
Yes	3	42.9%
No	4	57.1%
If infant was tested in first two months of life, what was HIV test result? (*N* = 3)		
Positive	0	0%
Negative	3	100%

Note: Data on missed opportunities related variables were collected from 40 study participants and 1 child was missing

Most caregivers of CLHIV enrolled in HIV services were satisfied with the enrollment experience (*n* = 29, 85%) and the information received about diagnosis and medical needs of the child (*n* = 31, 91%), although one caregiver recommended that more information could be provided on protecting their child from death. Among the 32 children started on ART, the majority of caregivers (*n* = 30, 94%) were satisfied with the experience of ART initiation; the remaining two caregivers had children that died after ART initiation.

Seven (17%) children were not linked to HIV services or initiated on ART during three months of follow-up; four were from the index testing entry point, two were from the malnutrition entry point, and one was from the TB clinic. Five were female, and four were 5 years of age or older. All seven had a living mother, while only five had a living father. Caregivers stated the following reasons for not linking to HIV services or initiating their child on ART: they preferred alternative methods of HIV treatment (*n* = 3, 43%), they did not see the need for services (*n* = 2, 29%), death before enrolling in HIV services (*n* = 2, 29%), they took their child to the facility but the child was not enrolled (*n* = 1, 14%), and lack of psychological readiness (*n* = 1, 14%). Some caregivers noted multiple reasons for not enrolling.

### Missed opportunities for earlier diagnosis

Only 4 (10%) mothers of the 40 newly diagnosed children had been diagnosed with HIV prior to or during pregnancy, and 3 (8%) mothers were diagnosed during breastfeeding (Table 4). Of these 7 women, only 3 (43%) had their infant tested within the first two months of life; all 3 reported negative infant tests. The majority of mothers of newly diagnosed children (*n* = 26, 65%) were diagnosed after the end of breastfeeding, and most of their children (78%) were ≥ 5 years at diagnosis. Three of the 26 (12%) women diagnosed after breastfeeding cessation had been offered testing during pregnancy, only 1 accepted. Timing of maternal HIV diagnosis was unknown in 7 children (18%).

### Disclosure of HIV status

Only 10 (24%) of the 41 CLHIV had been disclosed of their HIV status within the 3-month follow-up. When limiting the analysis to newly diagnosed children aged 10–14 years (*n* = 18), an age when disclosure of HIV status to the child can be expected, only 8 (44%) had been disclosed to during the 3-month follow-up period. Within the 10–14 age band, disclosure rates did increase with age, as 78% (7/9) of children aged 13–14 had been disclosed to, compared to only 1/9 (11%) children aged 10–12. Caregiver reasons for non-disclosure included: feeling the age was not appropriate (*n* = 23/31, 74%), didn’t know how to tell the child (*n* = 1, 3%), afraid to tell their child (*n* = 5, 16%), and afraid of psychologically traumatizing the child (*n* = 2, 6%).

## Discussion

To improve rates of linkage, same-day and early ART initiation have been promoted within the test and treat approach. However, data on timing of ART initiation in children in the test and treat era is limited. Confirmatory HIV testing has been recommended by the World Health Organization prior to ART initiation [6]; therefore, the timing of confirmatory testing greatly impacts the timing of ART initiation. However, there is little published literature on uptake and timing of confirmatory testing. Our study saw delays in conducting the confirmatory HIV test, with 17% not receiving confirmatory testing by 3 months. Although almost half of the children in our study that received confirmatory testing did so on the same day as initial diagnosis, almost one-third received it more than 1 week after initial diagnosis. As the majority of children initiated ART at the same facility as they received initial testing, delays in confirmatory testing did not appear to be due to referrals to another facility or caregiver preference for initiating ART at a different facility. Timing of confirmatory testing did not always correlate with timing of ART initiation, as only one-third of children received rapid ART initiation within 1 week of initial diagnosis. The rates of same-day ART initiation in our study (< 10%) were lower than a study in Rwanda comparing time to ART pre- and post-transition to “treat all” for children < 5 years (37%) [9]. Our study found that confirmatory testing seemed to make both caregivers and HCWs feel more comfortable with the child’s HIV diagnosis and ART initiation. Efforts are needed to ensure same-day or early confirmatory testing to increase rapid ART initiation CLHIV, and to decrease the time between confirmatory testing and ART initiation. This study did not collect data on reasons for delayed confirmatory testing or on implementation of services such as accompanied referral from the testing entry point to ART clinic; additional data on these may identify additional interventions to reduce time to confirmatory testing. Perceived challenges with confirmatory testing cited in our study, such as inadequate space or testing registers, can be addressed to ensure confirmatory testing is completed and is not a barrier to ART initiation. Post-test counseling for newly diagnosed children should ensure caregiver understanding of the need for timely confirmatory testing before ART initiation. Our study identified five (12%) deaths in CLHIV within three months of diagnosis amongst children not yet enrolled in HIV services, children enrolled but not yet initiated on ART, and children who had been initiated on ART. More intensive screening and support for pediatric advanced HIV disease at diagnosis is needed, to ensure rapid ART initiation and management of co-infections and comorbidities to prevent HIV-related mortality.

Reported linkage rates of enrollment into HIV services or ART initiation rates in CLHIV vary widely across studies (45–100%) [9–18], likely due to differing time points for follow-up assessments, different settings of HIV testing and diagnosis, and different age bands. For instance, one study from Cameroon found that linkage rates to HIV care or ART initiation were higher in index testing strategies compared to outpatient department (OPD) (85.0% vs. 52.5%) and in children ≤12 years compared to children 13–19 years [10]. Several studies were also conducted before [10–15] or during [16–18] the rollout of test and treat, which may have impacted enrollment and ART initiation rates. Another study in Cameroon also found that children aged 5–9 years, in primary school, and female were more likely to be enrolled on ART, but none of these were found to be statistically significant [16]. Even though the linkage rates to HIV services and ART initiation in our study are relatively high compared to other studies in CLHIV, they are not enough to meet UNAIDS 95–95-95 goals.

Many studies, including ours, did not capture or report details around strategies for supporting linkage. Several studies noted phone- or home-based tracing by community health workers for all newly diagnosed clients [11] or for clients that were not linked to HIV services [13, 17, 18]. The PoP-ART-Y study found high linkage rates in young adolescents 10–14 years (92.9% in boys, 98.2% in girls) with multiple active linkage interventions including escorts to clinics, specially-trained counselors, use of community health workers to track clients, and multi-disciplinary meetings to review those not yet linked to care or initiated on ART [19]. Another study from Kenya showed improved linkage rates in 10–14-year-old children after an adolescent-focused intervention that included capacity building, extended clinic hours, and program tools (53.8 to 83.6% for boys, 44.8 to 94.7% for girls) [20]. There is a need for better understanding scale of these types of comprehensive linkage interventions and their impact on younger children in order to achieve UNAIDS goals. In addition to active linkage strategies and early follow-up, there is a need to identify additional services to help caregivers with decision-making around treatment initiation for their children. HCWs should understand reasons for poor linkage and provide counseling that addresses common misconceptions and fears in the local context. Our study shows that in Ethiopia, more clarifying factual information is needed on perceived alternative treatments for HIV among caregivers, as well as enhanced treatment literacy and psychosocial support. Linkage to psychosocial services within or outside of ART clinic, such as through peer support groups, peer mentors/cadres, or OVC programs, may provide caregivers with additional education and support that can overcome these fears and misconceptions.

The rates of disclosure in our study were within the wider range of a recent systematic review that found prevalence of disclosure ranged from 9 to 72% among CLHIV in sub-Saharan Africa, with the rate increasing for older children [21]. However, most studies of disclosure in CLHIV report rates of disclosure in children already on ART aging into adolescence. Our study provides important data on rates of disclosure among newly diagnosed young adolescents. Disclosure is a process, and we would not expect rates of disclosure within 3 months of diagnosis to be at 100%, particularly in younger children; however, for younger adolescents, more rapid disclosure may be needed, as many may be mature and have access to online platforms to investigate their new medications. More data is needed to understand the impact of timing of disclosure on linkage to ART and other treatment outcomes. There is a need to intensify training for HCWs, including counsellors and case managers, on conducting disclosure counselling and addressing common challenges faced during the process, such as caregiver reluctance, fears, and lack of knowledge on optimal process and timing. Disclosure is an important component of adolescent-oriented HIV services to support linkage to ART and continuity of treatment. There are working documents substantiating the benefits of disclosure [23].

In our study, most late pediatric HIV diagnoses were due to lack of maternal HIV testing during pregnancy/breastfeeding; since the majority of children were older (i.e.,10–14 year), their mothers may not have had access to prevention of mother to child transmission (PMTCT). Among known HIV-positive mothers, early infant diagnosis (EID) uptake was poor, likely due to relatively lower coverage of PMTCT services when these children were born. Since the few infants receiving EID all had negative tests at two months of age, these children were likely infected during breastfeeding and missed follow-up testing. A study in Kenya evaluating the relative contribution of PMTCT gaps on missed testing opportunities of children of women living with HIV found that 31% of mothers had not been tested for HIV during pregnancy, and an additional 51% of mothers tested negative during pregnancy [17]. Their data from our study and the Kenya study align with a special analysis of the 2019 UNAIDS estimates showing a growing proportion of pediatric infections during the breastfeeding period (47% globally in 2018) [22]. The Kenya study also found similarly low rates of EID among HIV-exposed children: only 50% of children known to be HIV-exposed at birth had an HIV test before cessation of breastfeeding [17]. To avert pediatric infection and improve earlier identification of CLHIV, PMTCT and EID efforts need to be strengthened to ensure testing at all recommended time points for both the mother and infant, with increased focus on the breastfeeding period.

Strengths of this study include complete follow-up with all 41 caregivers three months after enrollment, which provides novel data on time to ART initiation in CLHIV in the era of “test and treat”, implementation of and perspectives on confirmatory HIV testing, and timing of disclosure in newly diagnosed CLHIV in Ethiopia. This sample size is similar to some studies from countries with higher HIV prevalence [10, 13, 16]. However, the small sample size of CLHIV identified and linked to ART limits the ability to conduct additional analysis to identify factors associated with linkage to HIV services and rapid ART initiation. Findings from this study are not nationally representative or generalizable due to the study design. Study findings are limited to those children whose caregivers consented for HIV testing, enrollment into the study, and follow-up. Data on specific services and interventions to support linkage was not captured, and services may have varied across study facilities and impacted linkage rates.

## Conclusions

There remain opportunities to strengthen linkage for newly diagnosed CLHIV in Ethiopia through intensifying early follow-up to ensure prompt confirmatory testing and ART initiation. Additional services could help caregivers with decision-making around timely confirmatory testing and ART initiation for their children. In settings such as ours, where high proportions of newly diagnosed CLHIV are older children and young adolescents, support for disclosure of HIV status to the child and linkage to adolescent-friendly services are important steps to support linkage to ART and continuity of treatment. To reduce pediatric infections and improve earlier identification of CLHIV, PMTCT and EID efforts need to be strengthened to ensure testing at all recommended time points for both the mother and infant, with increased focus on the breastfeeding period.
